# Downregulation of Caveolin-1 Enhances Fusion of Human BeWo Choriocarcinoma Cells

**DOI:** 10.1371/journal.pone.0010529

**Published:** 2010-05-06

**Authors:** Gavin P. Collett, Elizabeth A. Linton, Christopher W. G. Redman, Ian L. Sargent

**Affiliations:** Nuffield Department of Obstetrics and Gynaecology, University of Oxford, Oxford, United Kingdom; University of São Paulo, Brazil

## Abstract

**Background:**

Fusion of placental villous cytotrophoblasts with the overlying syncytiotrophoblast is essential for the maintenance of successful pregnancy, and disturbances in this process have been implicated in pathological conditions such as pre-eclampsia and intra-uterine growth retardation. Caveolin-1 has been shown to be expressed in human villous cytotrophoblast and to be downregulated during fusion into syncytiotrophoblast but it is unclear whether it plays a role in this process.

**Methodology/Principal Findings:**

We used RNA interference to determine whether caveolin-1 plays a role in differentiation and fusion in the BeWo choriocarcinoma cell line, a model of villous cytotrophoblast fusion. Assessment of cell fusion by desmosomal protein immunostaining revealed that cells transfected with caveolin-1 siRNA showed significantly enhanced fusion in response to treatment with dibutyryl cyclic AMP compared with cells transfected with a non-silencing control. Furthermore, caveolin-1 knockdown alone was sufficient to promote spontaneous fusion. In addition, biochemical differentiation, assessed by expression of placental alkaline phosphatase, was upregulated in caveolin-1 siRNA-transfected cells, with or without dbcAMP treatment. Assessment of Akt phosphorylation showed that caveolin-1 knockdown resulted in a significant reduction in phosphorylation at Thr^308^.

**Conclusions/Significance:**

Taken together, these results suggest that caveolin-1 regulates BeWo cell differentiation and fusion, possibly through a mechanism involving modulation of Akt activity.

## Introduction

Fusion of proliferating villous cytotrophoblast stem cells is essential to form, grow and maintain the syncytiotrophoblast epithelium of the human placenta [1]. Syncytiotrophoblast is in direct contact with maternal blood and serves key placental functions such as nutrient and gas exchange, and the synthesis of steroid and peptide hormones [2]. Disturbances in this process may be involved in pathological conditions such as pre-eclampsia and intra-uterine growth retardation [3], [4]. In studies of primate trophoblasts, it was discovered that syncytiotrophoblast formation originates as a result of fusion of mononuclear cytotrophoblasts [1]. Expansion of the syncytiotrophoblast throughout gestation then occurs as cytotrophoblasts proliferate, differentiate and finally fuse with the overlying syncytiotrophoblast. Therefore, trophoblast fusion is crucial for the beginning and maintenance of successful pregnancy. Indeed, it has been suggested that disturbances in this process may be involved in pathological conditions such as pre-eclampsia and intra-uterine growth retardation [3], [4]; however, the mechanism by which it occurs remains poorly understood. It has been shown that isolated cytotrophoblasts aggregate and fuse in vitro to form multinucleated syncytiotrophoblast [5] and this is enhanced by EGF or agents which increase intracellular cAMP levels. A number of proteins have been implicated in the fusion process, including envelope proteins derived from human endogenous retroviruses (HERVs) [6], caspase-8 [7], connexin 43 [8] and ADAM proteins [9].

Caveolin is the major structural component of caveolae, plasmalemmal organelles which function as macromolecular vesicular transporters and organizers of multiple signalling molecules [10]. Three caveolin isoforms are expressed in mammalian cells; caveolin-1 and caveolin-2 are co-expressed in most cell types whereas caveolin-3 is specifically expressed in muscle cells [11]. Although it was initially identified as a component of caveolae [11], caveolin-1 expression is not restricted to these organelles; it has been shown to localize to other cellular compartments including secretory vesicles, cytoplasm and mitochondria [12]. In its role as a scaffolding protein, caveolin-1 is capable of recruiting numerous signalling molecules as well as regulating their activity [13], [14]. Whilst many proteins, such as EGF receptor [15] and protein kinase C [16] are negatively regulated by direct interaction with caveolin-1 through its scaffolding domain, there are some instances where association with caveolin-1 is essential for activation, such as in adipocytes where interaction of the insulin receptor with caveolin-1 is required for successful insulin signaling [17].

Caveolin-1 is expressed in human cytotrophoblast and previous studies have revealed that cytotrophoblast differentiation and fusion appears to be associated with a marked depletion in detectable caveolin-1 levels [18]. However, it is not known whether this depletion is a cause or consequence of syncytial fusion. A possible role for caveolin in cell fusion has been demonstrated by the finding that manipulation of the levels of caveolin-3 in myoblasts affects the ability of these cells to fuse into multinucleated myotubes [19]. Therefore, in this study we have used RNA interference to knock down expression of caveolin-1 and assess its role in syncytial fusion in the BeWo choriocarcinoma cell line, a widely-used model of trophoblast fusion which expresses similar levels of caveolin-1 to those in native cytotrophoblasts [20].

## Materials and Methods

### Cell culture

BeWo cells obtained from the European Collection of Cell Cultures (Porton Down, UK) were cultured in full growth medium (Dulbecco's modified Eagle's medium/Ham's F12 supplemented with 2 mM l-glutamine, 100 IU/ml penicillin, 100 µg/ml streptomycin, 0.25 µg/ml amphotericin (Sigma) and 10% (v/v) fetal calf serum (Serum Laboratories International)). Cells were grown as a monolayer at a density of 10^7^ cells per 75 mm^2^ flask at 37°C in 95% air and 5% CO_2_, with medium changed every 48 h. For passages, cells were detached with trypsin/EDTA (Life Technologies) at 37°C, then washed in complete culture medium and replated.

### siRNA transfection

Cells were plated at 5×10^4^ cells/ml into 24-well plates and transfected immediately with caveolin-1 siRNA (sense 5′-GCCGUGUCUAUUCCAUCUA-3′; antisense 5′-UAGAUGGAAUAGACACGGC-3′) or a non-silencing negative control siRNA (sense 5′-UUCUCCGAACGUGUCACGU-3′; antisense 5′-ACGUGACACGUUCGGAGAA-3′) (Qiagen) using Hiperfect transfection reagent (Qiagen) following protocols provided by the manufacturer. After 48 h of incubation the cells were either harvested for protein extraction and immunoblotting or treated as described below for immunocytochemistry and analysis of cell fusion.

### Immunoblotting

Cells were washed with PBS and lysed directly into SDS–PAGE loading buffer. Protein concentration was determined using a BCA protein assay kit (Pierce). Proteins (5 µg) were resolved by SDS–PAGE and transferred to PVDF membrane. Membranes were then incubated in blocking buffer (Tris-buffered saline with 5% BLOTTO (Santa Cruz) and 0.1% Tween) for 45 minutes at room temperature then incubated with the appropriate primary antibodies in blocking buffer overnight at 4°C. Reactions were visualized by using a suitable secondary antibody conjugated to horseradish peroxidase (Dako) in blocking buffer at room temperature for 2 hours and an enhanced chemiluminescence system (Pierce). Primary antibodies used were: anti-caveolin-1 (#sc-894, Santa Cruz), anti-β-actin (#ab6276, Abcam), anti-phospho-Akt Thr^308^ (#2965), anti-phospho-Akt Ser^473^ (#4060), anti-Akt (#4691) (all New England Biolabs) and anti-placental alkaline phosphatase (NDOG2, Tannetta et al., 2008).

### Immunocytochemistry

Cells for desmosomal protein staining were fixed with ice-cold methanol and stored at 4°C in PBS until processing. For placental alkaline phosphatase staining, cells were fixed in 4% PFA in PBS, otherwise cells were permeabilised by incubation in PBS containing 0.5% Triton-X 100 (Sigma) for 10 min at room temperature. All cells were blocked for 1 h at room temperature with PBS containing 10% normal human serum and 0.1% Tween-20, then incubated overnight at 4°C with primary antibodies (anti-desmosomal protein (#D1286, Sigma), anti-placental alkaline phosphatase, see above) diluted in blocking buffer before washing (2×5 min) in PBS. Negative controls comprised mouse IgG alone. Cells were next stained with Alexa Fluor 488 conjugated secondary antibody (Invitrogen) in blocking buffer, the nuclei stained with Hoechst 33342 (1 µg/ml in PBS; Invitrogen) and washed (2×5 min) and stored in PBS. They were examined using a Leica DMIRE2 inverted fluorescence microscope and photographed using a Hamamatsu Orca monochrome camera and Simple PCI software (C Imaging).

### Cell fusion analysis

Cells were induced to fuse by replacing full growth medium with Dulbecco's modified Eagle's medium containing 2.5% (v/v) fetal calf serum and 1 mM dibutyryl cyclic AMP, as described previously [20]. At the indicated time points the cells were washed with PBS and fixed in methanol at −20°C for 10 minutes. Intercellular boundaries were then visualized by immunocytochemical staining with an antibody to desmosomal protein as described above. Ten random fields containing approximately 300 nuclei each were photographed for subsequent analysis. Composites of Hoechst 33342 stained nuclei and Alexa Fluor 488 stained desmosomal protein were made, then all nuclei were counted and the percentage of nuclei contained within multinucleated (≥2 nuclei) syncytia was calculated, as described previously [21].

### Statistical analysis

The data presented represent mean ± SEM of at least three separate experiments. Differences between control and caveolin-1 siRNA transfected cells were analysed by ANOVA and a *P*-value of less than 0.05 was considered to be statistically significant.

## Results

### siRNA-mediated downregulation of caveolin-1 expression in BeWo cells

**Figure 1:**
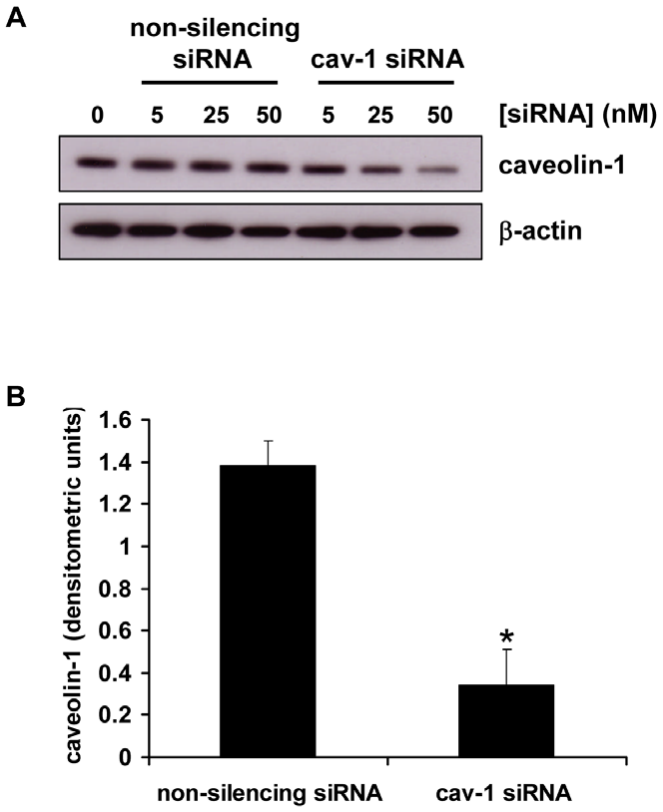
siRNA-mediated downregulation of caveolin-1 expression in BeWo cells. *A*, BeWo cells were transfected with the indicated concentrations of caveolin-1(Cav-1) siRNA or a non-silencing control siRNA. After 48 h cells were lysed and levels of caveolin-1 and β-actin were assessed by immunoblotting. *B*, densitometric analysis of immunoblots assessed for caveolin-1 expression and normalised to β-actin expression, in cells transfected for 48 h with 50 nM Cav-1 siRNA or non-silencing control siRNA. Results are presented as mean ± SEM for three separate experiments, *p<0.001 compared with control transfected cells (determined by ANOVA).

BeWo cells were transfected with siRNA duplexes targeted to human caveolin-1 mRNA (Cav-1 siRNA) or a non-silencing control which has no homology to any known mammalian gene. Caveolin-1 expression was efficiently downregulated in a dose-dependent manner by transfection (48 h) with Cav-1 siRNA, as demonstrated by an immunoblot of BeWo cell lysates (Fig. 1A). Transfection with the non-silencing control did not affect caveolin-1 expression compared with mock-transfected controls. Levels of β-actin were unaffected by transfection with either Cav-1 or non-silencing siRNA. Densitometric analysis revealed that transfection with 50 nM Cav-1 siRNA resulted in a 72% knockdown of caveolin-1 expression compared with the non-silencing control (Fig. 1B). For all further experiments, cells were transfected with siRNA duplexes at 50 nM.

### Caveolin-1 knockdown enhances BeWo cell fusion

**Figure 2:**
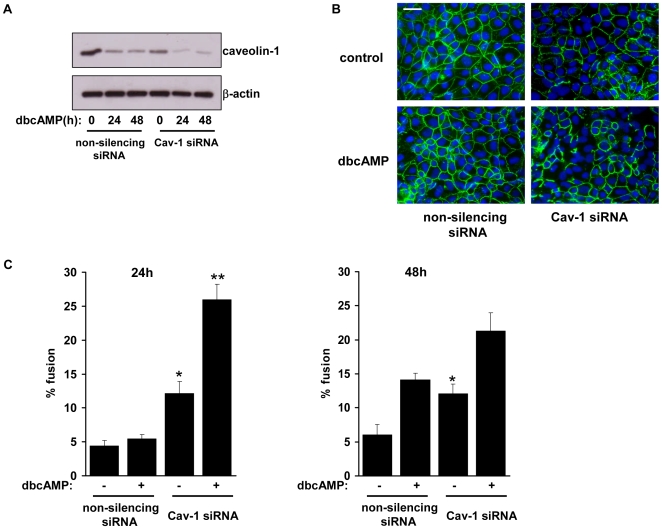
Assessment of cell fusion. *A*, BeWo cells were transfected with Cav-1 siRNA or non-silencing control siRNA. After 48 h the cells were treated with low (2.5%) serum medium containing 1 mM dbcAMP. At the indicated timepoints, cells were lysed and levels of caveolin-1 and β-actin were determined by immunoblotting. *B*, Cells were transfected as in *A* then treated with low (2.5%) serum medium in the absence of dbcAMP (1 mM), as indicated, for 24 h. Cells were fixed, immunostained for desmosomal protein (green) and counterstained with Hoechst (blue). *C*, cells were treated as in *B* and cell fusion was quantified at 24 h (left panel) and 48 h (right panel) as described in Materials Methods& . Results are presented as mean ± SEM for three separate experiments, *p<0.001, **p<0.05 compared with control transfected cells (determined by ANOVA). Scale bar = 100 µm.

The effect of caveolin-1 knockdown on BeWo cell fusion was assessed by transfecting cells with Cav-1 or control siRNA for 48 h then treating with low-serum medium in the presence or absence of 1 mM dbcAMP, a cell-permeable analogue of cyclic AMP. Treatment with dbcAMP resulted in a decrease in Cav-1 expression and the silencing effect of Cav-1 siRNA was maintained over the 48 h treatment period, i.e. 96 h in total after the commencement of transfection (Figure 2A). To assess fusion, intercellular boundaries were visualised by staining with an antibody to desmosomal protein [22]. Figure 2B shows that after 24 h of treatment there was little evidence of cell fusion in control transfected cultures, as shown by the presence of intercellular boundaries, whereas in cells transfected with Cav-1 siRNA there were clear areas of multinucleated syncytia where the intercellular boundaries had been lost. Cell fusion was quantified by calculating the percentage of nuclei contained within multinucleated syncytia [21]. Without dbcAMP, Cav-1 siRNA-transfected cells showed a significant increase in the percentage of nuclei contained in syncytia compared with untreated cells transfected with non-silencing control (12.1% vs 4.4%, and 12.1% vs 6.1%, at 24 h and 48 h respectively; Fig. 2C). Similarly, with dbcAMP, the number of nuclei in syncytia was significantly increased in Cav-1 siRNA-transfected cells after 24 h treatment compared with controls (26.0% vs 5.4%). There was also an increase at 48 h (21.3% vs 14.1%) but this was not statistically significant.

### Placental alkaline phosphatase expression is upregulated by caveolin-1 knockdown

**Figure 3:**
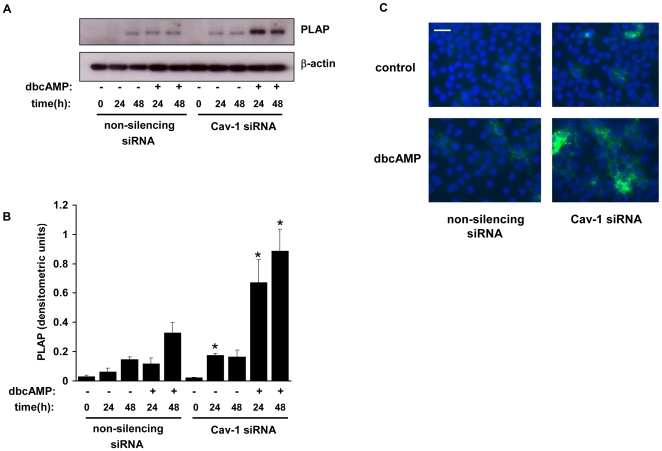
Assessment of placental alkaline phosphatase expression. *A*, BeWo cells were transfected with Cav-1 siRNA or non-silencing control siRNA. After 48 h the cells were treated with low (2.5%) serum medium in the presence or absence of dbcAMP (1 mM). At the indicated timepoints (0 h being equivalent to 48 h treatment with siRNA) cells were lysed and levels of PLAP and β-actin were determined by immunoblotting. *B*, densitometric analysis of immunoblots showing the level of PLAP, normalised to β-actin levels, in Cav-1 and control transfected cells. *C*, cells were treated as in *A*. After 24 h the cells were fixed, immunostained for PLAP (green) and counterstained with Hoechst (blue). Results are expressed as mean ± SEM for three separate experiments, *p<0.05 compared with control transfected cells (determined by ANOVA). Scale bar = 100 µm.

Differentiation and fusion of cytotrophoblasts into syncytia is accompanied by a strong upregulation of placental alkaline phosphatase (PLAP) expression [23]. Therefore we assessed the effect of caveolin-1 knockdown on PLAP expression in BeWo cells. Immunoblotting showed that in proliferating, undifferentiated BeWo cells PLAP levels were extremely low but were increased by 24 h following treatment with low-serum medium containing dbcAMP, and by 48 h after treatment with low-serum medium alone (Fig. 3A). In cav-1 siRNA transfected cells PLAP expression upon dbcAMP treatment was significantly enhanced at both 24 h and 48 h compared with control cells (Figs. 3A & 3B). Furthermore, following treatment with low-serum medium alone, PLAP expression was significantly higher at 24 h in caveolin-1 siRNA transfected cells. These results were confirmed by immunocytochemical studies, which revealed an increase in PLAP expression at 24 h in caveolin-1 siRNA transfected cells compared with control cells, following treatment in low-serum medium with or without dbcAMP (Fig. 3C).

### Akt phosphorylation is inhibited by caveolin-1 knockdown

**Figure 4:**
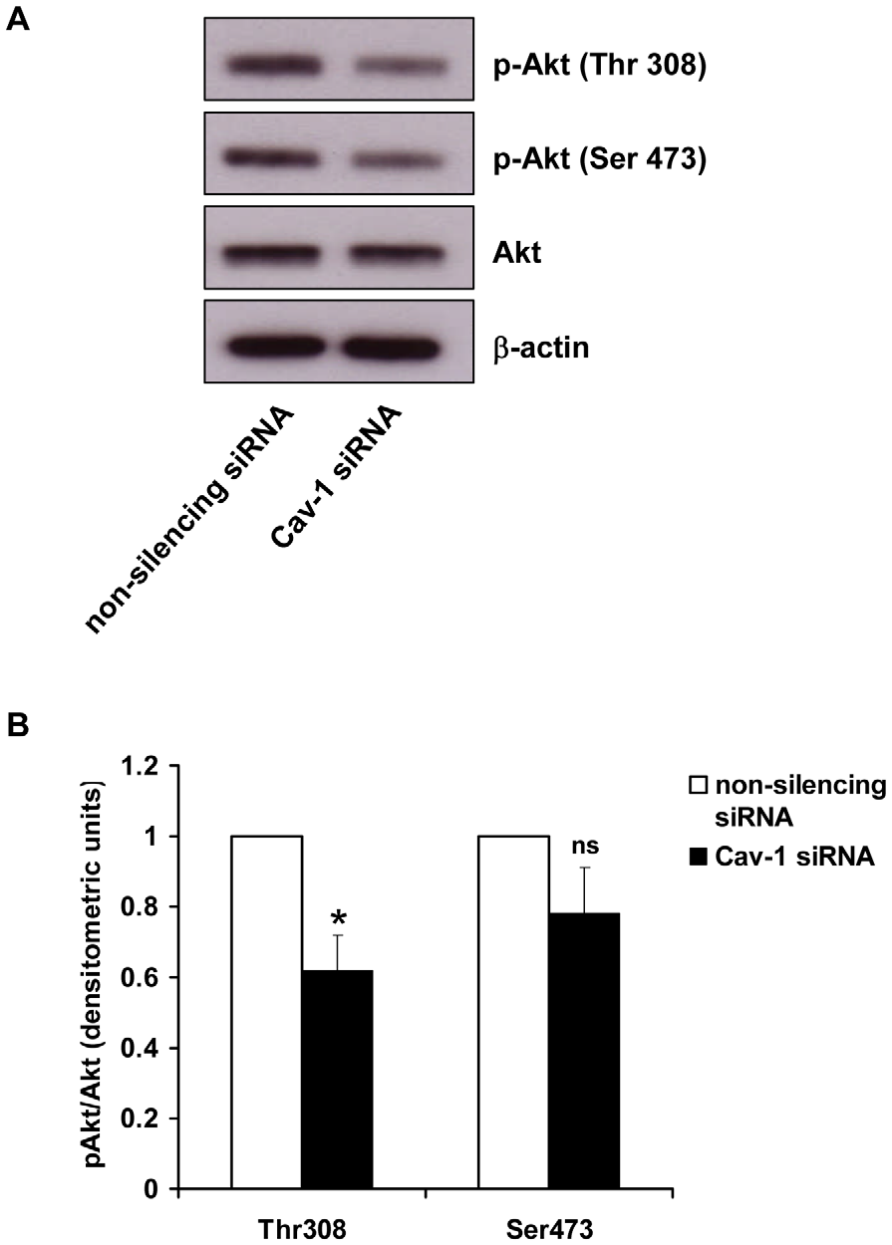
Effect of caveolin knockdown on Akt phoshorylation. *A*, BeWo cells were transfected with Cav-1 siRNA or non-silencing control siRNA. After 48 h the cells were lysed and levels of phosphorylation of Akt at Thr^308^ and Ser^473^ were assessed by immunoblotting using phosphorylation site-specific antibodies. β-actin and total Akt levels were determined as loading controls. *B*, densitometric analysis of immunoblots showing the level of Akt Thr^308^ and Ser^473^ phosphorylation, normalised to total Akt levels, in Cav-1 and control transfected cells. Results are expressed as mean ± SEM for four separate experiments, *p<0.01 compared with control transfected cells (determined by ANOVA). ns = non-significant.

Akt has been shown to play a role in myoblast fusion [24] and, in other cell types, is regulated by caveolin-1 [25], [26], [27]. Akt is fully activated after phosphorylation at Thr^308^ and Ser^473^ by PDK1 (phosphoinositide-dependent protein kinase 1) or mTORC2 (mammalian target of rapamycin complex 2) [28]. Therefore, to explore the possibility that caveolin may regulate Akt activity in trophoblasts we used phosphorylation site-specific antibodies to study the activation status of Akt in BeWo cells transfected with control or Cav-1 siRNA. Immunoblots revealed that in control transfected cells Akt was phosphorylated at both Thr^308^ and Ser^473^ (Fig. 4A). In cells transfected with Cav-1 siRNA there was a reduction in Thr^308^ phosphorylation (Fig. 4A) relative to control cells. Total Akt levels were unaffected by caveolin-1 knockdown. Densitometric analysis showed that Thr^308^ phosphorylation was significantly reduced by 38% in Cav-1 siRNA transfected cells (Fig. 4B). No significant difference in Ser^473^ phosphorylation was observed in Cav-1 siRNA transfected cells compared to controls (Figs 4A & B).

## Discussion

In this study we show for the first time that downregulation of caveolin-1 enhances differentiation and fusion in the BeWo human choriocarcinoma cell line, as assessed using both morphological and biochemical criteria. These results suggest that caveolin-1 may play an important role in the regulation of these processes in normal and/or pathological pregnancies.

We have used BeWo cells as a model of trophoblast differentiation and fusion. This is a well characterised model which shares important properties with freshly isolated human villous cytotrophoblasts, most significantly the ability to fuse and form large multinucleated syncytia [29]. Mechanistic aspects of intercellular fusion, such as externalisation of phosphatidylserine [30] and upregulation of syncytin-1 expression [31], [32] have been shown to be common to both cell types. Importantly, in the context of this study, BeWo cells and primary villous cytotrophoblasts express similar levels of caveolin-1 [20] suggesting that the results obtained from knockdown of the protein in BeWo cells as described here will be pertinent to understanding its role in trophoblast fusion in placental development.

Cell fusion to form syncytia is a rare event in cell biology and is restricted to striated muscle fibres, chondro- or osteoclasts, macrophage giant cells and syncytiotrophoblast [3], [33]. Whilst no data prior to this study exist on a role for caveolin in trophoblast fusion, it has been demonstrated that the ability of myoblasts to fuse to form multinucleated myotubes is affected by their level of muscle-specific caveolin-3, although it is unclear as to whether it enhances or inhibits fusion. In mice it has been shown that overexpression of caveolin-3 inhibits myoblast fusion whereas the process is enhanced by a lack of caveolin-3 [19]. However, more recent studies in zebrafish have suggested that caveolin-3 deficiency may lead to a reduction in myoblast fusion [34]. In the human placenta, caveolin-1 is strongly expressed in endothelial cells, with lower levels in cytotrophoblasts and mesenchymal cells, and barely detectable expression in syncytiotrophoblast [35], [36], [37]. This reduction in expression of caveolin-1 in syncytiotrophoblast compared to cytotrophoblast, together with the observation that differentiation and fusion of cytotrophoblast in vitro is accompanied by its depletion [18], led us to hypothesise that it may play an important role in these processes. In the present study, downregulation of caveolin-1 by RNA interference resulted in an augmentation of dbcAMP-induced cell fusion, and also induced fusion in the absence of dbcAMP. We found that, in control cells, dbcAMP had no effect at 24 h but induced fusion at 48 h. At both timepoints fusion was enhanced in caveolin-1 siRNA-transfected cells compared with control cells following dbcAMP treatment. Furthermore, in the absence of dbcAMP, caveolin-1 siRNA transfection resulted in a marked and significant increase in syncytium formation at both 24 h and 48 h, demonstrating that caveolin-1 downregulation alone is sufficient to promote trophoblast fusion. Interestingly, no further increase in the fusion rate in caveolin-1 siRNA-transfected cells from 24 h to 48 h was observed. This was not due to a lessening of the silencing effect over time, since it was clear that caveolin-1 expression was maintained at lower levels in caveolin-1 siRNA-transfected cells even after 48 h of treatment with dbcAMP, i.e. 96 h after the commencement of transfection. The reason for this effect is therefore unclear but it is possible that the lack of increase in observed fusion from 24 h to 48 h may be explained by death and/or sloughing off of some fused cells from the tissue culture plate at the later time point. Higher rates of fusion were observed after a combination of caveolin-1 knockdown and dbcAMP treatment than with either condition alone, suggesting that both conditions might be acting to enhance a common signalling pathway; it is also possible, however, that caveolin-1 downregulation may act to stimulate fusion by a mechanism which is distinct from that elicited by cyclic AMP. In addition to its effect on fusion we also looked at the impact of caveolin-1 knockdown on biochemical differentiation of BeWo cells. Although PLAP cannot be regarded as a bona fide marker of syncytiotrophoblast, since it is expressed at low levels in some cytotrophoblasts, it is clearly upregulated during trophoblast differentiation and fusion [23]. We found that caveolin-1 knockdown enhanced PLAP expression in dbcAMP-treated cells, whilst in the absence of dbcAMP, caveolin-1 downregulation resulted in a more rapid expression of PLAP compared with non-silencing controls. These results show that cav-1 knockdown enhances biochemical differentiation of BeWo cells, mirroring its effects on morphological differentiation described in the fusion experiments.

The mechanism by which caveolin-1 regulates BeWo cell fusion remains to be elucidated. In myoblasts, Akt activity has been shown to be required for differentiation into myotubes and their subsequent survival and growth [24]. Since Akt is expressed by trophoblast [38] and, in other cell types, can be either positively or negatively regulated by caveolin-1 [25], [26], [27], we decided to investigate the effect of caveolin-1 knockdown on Akt activation in trophoblast using phosphorylation site-specific antibodies. Full activation of Akt requires phosphorylation at both Thr^308^ and Ser^473^ [28]. We found that Akt was constitutively phosphorylated in BeWos at Thr^308^ and Ser^473^ and that downregulation of caveolin-1 resulted in a significant reduction of phosphorylation at Thr^308^. A reduction in Ser473 phosphorylation in caveolin-1 knockdown cells was also observed but this was not found to be statistically significant. Interestingly, it has been demonstrated that in differentiating myoblasts Akt Thr^308^ phosphorylation is downregulated [24], and that dephosphorylation at this site leads to an alteration in the activity of a specific subset of Akt target proteins [39]. One such protein is the FoxO1 forkhead transcription factor family member, which has been shown to regulate myoblast fusion [40]. Furthermore, dephosphorylation of Akt is catalysed by protein phosphatases PP1 and PP2A [41], [42], [43] and, intriguingly, it has been shown that caveolin-1 can interact with both these phosphatases and negatively regulate their activity [25]. It is tempting, therefore, to speculate that the effects of caveolin on BeWo cell fusion may be mediated through its regulation of phosphorylation of Akt and subsequent downstream events. These themes will be explored in future work.

In conclusion, we show for the first time that the regulation of caveolin-1 expression could represent an important step in BeWo cell differentiation and fusion. Experiments in isolated primary human cytotrophoblasts will allow us to further elucidate the role of this protein in human trophoblast function.
